# Comparative chloroplast genomics and phylogenetics of nine *Lindera* species (Lauraceae)

**DOI:** 10.1038/s41598-018-27090-0

**Published:** 2018-06-11

**Authors:** Mei-Li Zhao, Yu Song, Jun Ni, Xin Yao, Yun-Hong Tan, Zeng-Fu Xu

**Affiliations:** 1 Center for Integrative Conservation, Xishuangbanna Tropical Botanical Garden, Chinese Academy of Sciences, Menglun, Mengla, Yunnan 666303 China; 2 Key Laboratory of Tropical Plant Resources and Sustainable Use, Xishuangbanna Tropical Botanical Garden, Chinese Academy of Sciences, Menglun, Mengla, Yunnan 666303 China; 3 College of Life Sciences, University of Chinese Academy of Sciences, Beijing, 100049 China; 4 Southeast Asia Biodiversity Research Institute, Chinese Academy of Sciences, Yezin, Nay Pyi Taw Myanmar

## Abstract

*Lindera*, a core genus of the Lauraceae family, has important economic uses in eastern Asia and North America. However, its historical diversification has not been clarified. In this study, we report nine newly sequenced *Lindera* plastomes. The plastomes of these nine *Lindera* species range from 152,211 (*L*. *nacusua*) to 152,968 bp (*L*. *metcalfiana*) in length, similar to that of another Lauraceae species, *Litsea glutinosa* (152,618 bp). The length variation of these plastomes derived from the length variation in the loci *ycf1*, *ycf2*, *ψycf1*, and *ndhF*-*ψycf1*. Comparing our sequences with other available plastomes in the Lauraceae indicated that eight hypervariable loci, *ihbA*-*trnG*, *ndhA*, *ndhF*-*rpl32*, *petA*-*psbJ*, *psbK*-*psbI*, *rps16*, *trnS*-*trnG*, and *ycf1*, could serve as DNA barcodes for species delineation, and that the inverted repeats (IRs) showed contraction/expansion. Further phylogenetic analyses were performed using 32 complete plastomes of Lauraceae and seven barcodes from 14 additional species of *Lindera* and related species in the core Lauraceae. The results showed that these *Lindera* species grouped into two or four sub-clades, and that two *Litsea* species and *Laurus nobilis* were located in the same sub-clade as five *Lindera* species. These data support a close relationship between the genera *Laurus*, *Lindera*, and *Litsea*, and suggest that *Lindera* is polyphyletic.

## Introduction

In plants, the chloroplast is the main locus of photosynthesis and carbon fixation^1^. The chloroplast (cp) genome ranges from 120 to 180 kb in size and has a characteristic structure, in which two inverted repeat (IR) regions divide the cp genome into four parts: the IRs themselves, a large single copy region (LSC) and a small single copy region (SSC)^2^. In the LSC, three of the four core plant barcodes, *psbA*-*trnH*, *rbcL*, and *matK*, have been widely used for identification purposes and phylogenetic analyses in the past twenty years^3^. Currently, complete cp genomes as well as full-length cp gene sequences are available for an increasing number of taxa, and both have been the subjects of numerous phylogenies. Phylogenomics, a technique for estimating phylogenetic relationships based on high-throughput sequencing, can allow a comprehensive understanding of the evolutionary history of organisms. For instance, Ma, *et al*.^4^ used cp phylogenomics to resolve the deep-level relationships of Arundinarieae. Yang, *et al*.^5^ used complete cp genome sequences to infer phylogenetic relationships in the genus *Quercus*. Very recently, Zhang, *et al*.^6^ provided important insights into deep phylogenetic relationships and the diversification history of the Rosaceae based on analyzing plastid phylogenomics.

Like Arundinarieae and Rosaceae, Lauraceae are a large monophyletic group, comprising approximately 3,500 known species from over 50 genera worldwide^7,8^, which are by far the largest family of the order Laurales^9^. The phylogenetic backbone is well resolved by now, but problems remain within most major clades^10–13^. Multiple classification schemes based on a variety of morphological and anatomical characteristics have been proposed, but none has been fully accepted. Therefore, other sources of data, such as genomic information, are needed for classification. As a foundation for further studies of Lauraceae phylogenomics, here we focus on *Lindera*^14^, a genus belonging to the core Lauraceae in the sense of Rohwer and Rudolph^15^, or the core Laureae in the sense of Chanderbali, *et al*.^11^ *Lindera* species have not only popular ornamental and economic uses but also great medicinal and therapeutic value. *Lindera* is widely distributed in tropical, subtropical, and temperate zones in Asia and North America and includes approximately 100 species^16^, with *Lindera umbellata* Thunb. as the type species. The fruits of most *Lindera* species, particularly *Lindera communis* Hemsley and *Lindera glauca* (Siebold & Zucc.) Blume, are rich in fatty oils and thus represent important wild woody oil plants^17–19^. *Lindera megaphylla* Hemsley is an economically important small deciduous tree, the wood of which can be used for buildings and furniture. Moreover, it is also a courtyard greening species, and its trunk and leaves are rich in alkaloids^20,21^. More importantly, *Lindera* plants are widely used in traditional medicine. *Lindera obtusiloba* Blume has been used as a traditional medicine for the treatment of fever, abdominal pain, extravasation, inflammation and poor blood circulation^22–25^. Thus, molecular methods for species delineation in the genus of *Lindera* are of considerable interest.

The first reported cp genomic markers in the Lauraceae were *rbcL* and *trnL*-*trnF*, which were used for phylogenetic analysis of the Laurales^9,26^. The *matK* gene was used to construct a phylogenetic tree to analyse the relationships among Lauraceae genera^10^, but in this analysis the Laureae (represented by a single species of *Actinodaphne*, *Laurus*, *Lindera*, *Litsea* and *Neolitsea*) remained unresolved. Then, Chanderbali, *et al*.^11^ constructed larger phylogenetic trees for the Lauraceae using the chloroplast sequences *trnL*-*trnF*, *psbA*-*trnH*, *trnT*-*trnL*, and *rpl16* as well as the nuclear barcoding markers 26S rDNA and internal transcribed spacer (ITS) rDNA. The result of their *trnL*-*trnF* + *psbA*-*trnH* analysis showed *Lindera erythrocarpa* as sister to *Litsea glaucescens*, albeit without bootstrap support, whereas in their ITS analysis *Lindera erythrocarpa* appeared as sister to *Laurus nobilis*, likewise without bootstrap support. Li, *et al*.^12^ and Nie, *et al*.^27^, who used ITS plus different chloroplast markers, found that the genus *Lindera* was not monophyletic. Fijridiyanto and Murakami^28^ further analysed the phylogenetic relationships of *Litsea* and its related genera using the nuclear marker *rpb2*, also finding that *Lindera* was not a monophyletic group. Most recently, a report^29^ showed a close relationship between the genera *Lindera* and *Litsea* while using *rbcL*, *matK*, *trnH–psbA*, and ITS to investigate the phylogenetic relationships in the Lauraceae.

In this study, we report the complete chloroplast genome sequences of nine *Lindera* species chosen for their economic importance. *Lindera communis*, *L*. *glauca*, *Lindera latifolia* Hook. f.^14^, and *Lindera nacusua* (D. Don) Merr^14^ are important wild woody oil plants, *L*. *megaphylla* and *Lindera robusta* (C. K. Allen) H. P. Tsui^14^ are important timber plants, and *Lindera benzoin* (L.) Blume^30^, *Lindera metcalfiana* var. dictyophylla (C. K. Allen) H. P. Tsui^14^, and *L*. *obtusiloba* are widely used in traditional medicine. In addition, compared to other Lauraceae species, *L*. *benzoin* and *L*. *obtusiloba* are distributed in more northern areas, and their wide distribution shows the ability to adapt to cold environments. Based on cp sequence information, the characteristics and phylogenetic information of these species were further investigated.

## Results

### Characteristics of the cp genomes of *Lindera*

**Figure 1:**
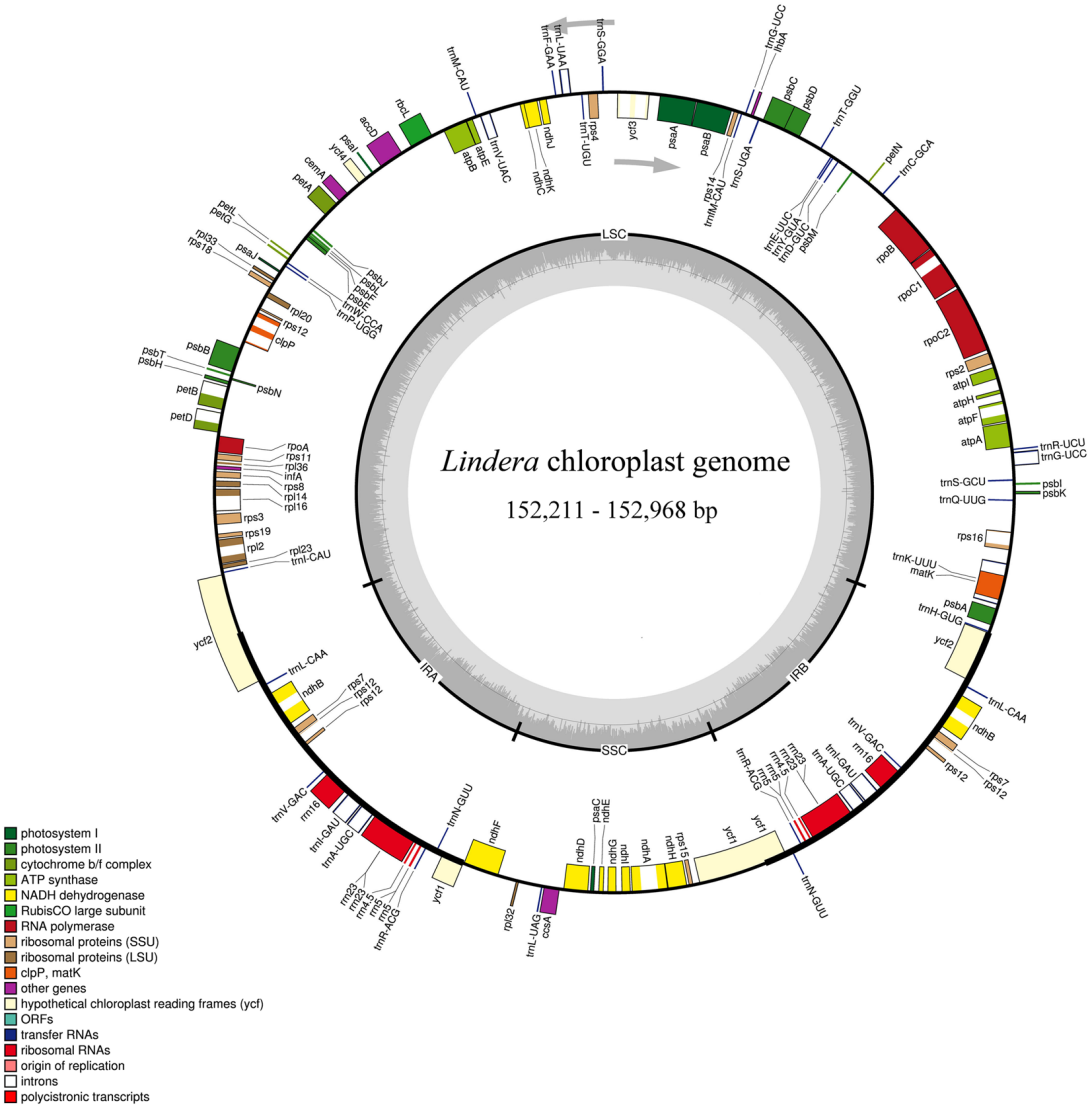
Circular gene map of *Lindera* species (*Lindera benzoin*, *L*. *communis*, *L*. *glauca*, *L*. *latifolia*, *L*. *megaphylla*, *L*. *metcalfiana*, *L*. *nacusua*, *L*. *obtusiloba*, and *L*. *robusta*) chloroplast genomes. The genes lying outside each circle are transcribed counter-clockwise, while those inside are transcribed clockwise. The coloured bars indicate different functional groups. The dashed darker grey area in the inner circle indicates genome GC content, while the lighter grey area shows AT content. IR = inverted repeat; SSC = small single copy; LSC = large single copy.

The sizes of the cp genomes of the nine *Lindera* species range from 152,211 (*L*. *nacusua*) to 152,968 bp (*L*. *metcalfiana*) (Table 1). The sequences were assembled into a single, circular, double-stranded DNA sequence for each species. The cp genomes have a typical quadripartite structure, comprising the LSC, with a length of 93,573 (*L*. *benzoin*) to 93,888 bp (*L*. *metcalfiana*), the SSC, with a length of 18,336 (*L*. *nacusua*) to 18,978 bp (*L*. *metcalfiana*), and a pair of IR copies of 20,048 (*L*. *benzoin*) to 20,075 bp (*L*. *obtusiloba*) in length (Fig. 1 and Table 1). The cp sequences of *Lindera communis*, *L*. *glauca*, *L*. *latifolia*, *L*. *megaphylla*, *L*. *metcalfiana*, *L*. *obtusiloba*, and *L*. *robusta* are larger than that of *Litsea glutinosa* (152,618 bp, GenBank accession number KU382356)^8^, but shorter than those of *Phoebe omeiensis* and *P*. *sheareri* (152,855 bp, GenBank accession number KX427772; 152,876 bp, GenBank accession number KX427773)^31,32^. The cp sequences of *Lindera benzoin* and *L*. *nacusua* are all shorter than those of *Litsea glutinosa*, *Phoebe omeiensis* and *P*. *sheareri* (Table 1).

**Table 1:** Summary of nine complete plastomes of *Lindera*.

	*L. benzoin*	*L. communis*	*L. glauca*	*L. latifolia*	*L. megaphylla*	*L. metcalfiana*	*L. nacusua*	*L. obtusiloba*	*L. robusta*
Total cpDNA size (bp)	152,478	152,778	152,706	152,779	152,711	152,968	152,211	152,773	152,852
Length of large single copy (LSC) region (bp)	93,573	93,748	93,650	93,792	93,651	93,888	93,735	93,714	93,860
Length of inverted repeat (IRs) region (bp)	20,048	20,066	20,054	20,070	20,066	20,051	20,070	20,075	20,061
Length of small single copy (SSC) region (bp)	18,809	18,898	18,948	18,847	18,928	18,978	18,336	18,909	18,870
Total GC content (%)	39.2	39.2	39.2	39.2	39.2	39.2	39.2	39.1	39.2
LSCGC content (%)	38	38	37.9	38	38	38	38	38	37.9
IRGC content (%)	44.5	44.4	44.5	44.4	44.4	44.5	44.4	44.4	44.5
SSCGC content (%)	34	34	33.8	33.9	33.9	34	34.1	33.8	34
Total number ofgenes	113	113	113	113	113	113	113	113	113
Total number of protein encodinggenes	79	79	79	79	79	79	79	79	79
Total number of tRNA	30	30	30	30	30	30	30	30	30
Total number of rRNA	4	4	4	4	4	4	4	4	4

All nine *Lindera* cp genomes contain 113 single-copy genes, among which 79 encode proteins. Sixteen genes have one intron (*atpF*, *ndhA*, *ndhB*, *petB*, *petD*, *rpl2*, *rpl16*, *rpoC1*, *rps12*, *rps16*, *trnA*-*UGC*, *trnG*-*UCC*, *trnI*-*GAU*, *trnK*-*UUU*, *trnL*-*UAA* and *trnV*-*UAC*), and two genes have two introns (*clpP* and *ycf3*) (Table 2). The *ycf1* and *ycf2* genes are trans-spliced, and the nucleotide sequences of the *ycf1* and *ycf2* pseudogenes are 1,372, 1,373, 1,376, 1,377, 1,379, 1,383, and 1,389 bp (*Lindera communis*, *L*. *megaphylla*, *L*. *metcalfiana*, *L*. *latifolia and L*. *nacusua*, *L*. *robusta*, *L*. *glauca*, *L*. *benzoin*, *and L*. *obtusiloba*), and 3,162 bp (all nine *Lindera* species), respectively, being truncated at the IR boundaries (Fig. 1). Additionally, the *ycf1*5 gene, located in the LSC of the nine *Lindera* genomes, is also a pseudogene (Table 2). The GC content of these *Lindera* cp genomes is 39.2%, except for *L*. *obtusiloba* (39.1%), which is similar to that of *Litsea glutinosa* (39.2%), but slightly higher than those of *Phoebe omeiensis* and *P*. *sheareri* (39.1%). The GC content of *L*. *obtusiloba* is the same as those of *Phoebe omeiensis* and *P*. *sheareri*^32^ (Table 1).

**Table 2:** Genes encoded by nine *Lindera* plastomes.

Category for genes	Group of genes	Name of genes
Photosynthesis related genes	Rubisco	*rbcL*
	Photosystem I	*psaA*, *psaB*, *psaC*, *psaI*, *psaJ*
	Assembly/stability of photosystem I	**ycf3, *ycf4*
	Photosystem II	*psbA*, *psbB*, *psbC*, *psbD*, *psbE*, *psbF*, *psbH*, *psbI*, *psbJ*, *psbK*, *psbL*, *psbM*, *psbN*, *psbT*, *ihbA*
	ATP synthase	*atpA*, *atpB*, *atpE*, *atpF, *atpH*, *atpI*
	cytochrome b/f compelx	*petA*, *petB, *petD, *petG*, *petL*, *petN*
	cytochrome c synthesis	*ccsA*
	NADPH dehydrogenase	*ndhA, *ndhB, *ndhC*, *ndhD*, *ndhE*, *ndhF*, *ndhG*, *ndhH*, *ndhI*, *ndhJ*, *ndhK*
Transcription and translation related genes	transcription	*rpoA*, *rpoB*, *rpoC1, *rpoC2*
	ribosomal proteins	*rps2*, *rps3*, *rps4*, *rps7*, *rps8*, *rps11*, *rps12*, *rps12, *rps14*, *rps15*, *rps16, *rps18*, *rps19*, *rpl2, *rpl14*, *rpl16, *rpl20*, *rpl23*, *rpl32*, *rpl33*, *rpl36*
	translation initiation factor	*infA*
RNA genes	ribosomal RNA	*rrn4*.*5*, *rrn5*, *rrn16*, *rrn23*
	transfer RNA	*trnA-*UGC*, *trnC*-*GCA*, *trnD*-*GUC*, *trnE*-*UUC*, *trnF*-*GAA*, *trnG*-*UCC*, *trnG-*UCC*, *trnH*-*GUG*, *trnI*-*CAU*, *trnI-*GAU*, *trnK-*UUU*, *trnL*-*CAA*, *trnL-*UAA*, *trnL*-*UAG*, *trnfM*-*CAU*, *trnM*-*CAU*, *trnN*-*GUU*, *trnP*-*UGG*, *trnQ*-*UUG*, *trnR*-*ACG*, *trnR*-*UCU*, *trnS*-*GCU*, *trnS*-*GGA*, *trnS*-*UGA*, *trnT*-*GGU*, *trnT*-*UGU*, *trnV*-*GAC*, *trnV-*UAC*, *trnW*-*CCA*, *trnY*-*GUA*
Other genes	RNA processing	*matK*
	carbon metabolism	*cemA*
	fatty acid synthesis	*accD*
	proteolysis	**clpP
Genes of unknown function	conserved reading frames	*ycf1*, *ycf2*
Pseudogenes		*ycf15*

Note: Asterisks (*) before gene names indicate intron containing genes, and double asterisks (**) indicate two introns in the gene.

### Identification of the most variable regions

**Figure 2:**
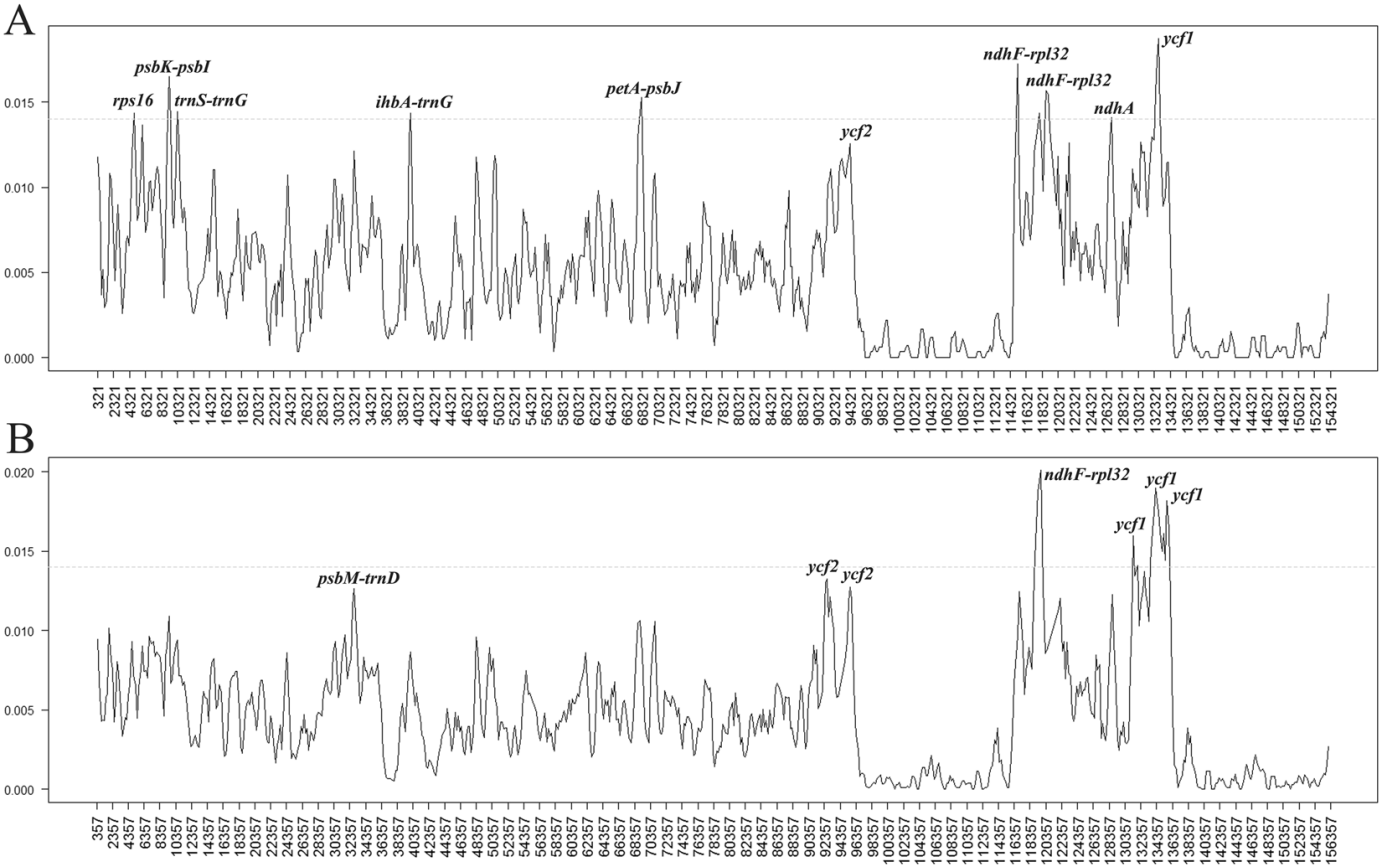
Comparision of the nucleotide variability (Pi) values of the nine *Lindera* plastomes (**A**) and 29 plastomes of the core Lauraceae (**B**). X axis: position of the midpoint of a window, Y axis: nucleotide diversity of each window.

To elucidate levels of sequence divergence, we calculated the nucleotide variability (Pi) values. The Pi values within 600 bp across the nine genomes vary from 0 to 0.0187, and the mean value is 0.0048 (Fig. 2A), indicating that these sequences have high similarity. However, we identified nine hypervariable loci (Pi > 0.014), which are *ihbA*-*trnG*, *ndhA*, *ndhF*-*rpl32*, *petA*-*psbJ*, *psbK*-*psbI*, *rps16*, *trnS*-*trnG*, and *ycf1*. The *ndhA* and *ndhF*-*rpl32* loci are in the SSC region; *ihbA*-*trnG*, *petA*-*psbJ*, *psbK*-*psbI*, *rps16*, and *trnS*-*trnG* are in the LSC region; and *ycf1* is in the IR region (Fig. 2A). To investigate the levels of sequence divergence among the genera, we calculated the genetic divergence of the sequenced cp genomes of core Lauraceae, including *Alseodaphne*, *Cinnamomum*, *Laurus*, *Lindera*, *Litsea*, *Machilus*, *Nectandra*, *Persea*, *Phoebe*, and *Sassafras*. The Pi values vary from 0 to 0.0201 in these 29 sequences (Fig. 2B), indicating that the variation at genus level is significantly larger than that at the species level. All these genomic features are shown in the sequence alignment of the nine *Lindera* species, *Laurus nobilis*, and *Litsea glutinosa* (Fig. S1). According to the alignment results (Fig. S1), all of these species share the same order and orientation of syntenic blocks, indicating that no rearrangement occurred in gene organization. These results accord with those of Male, *et al*.^33^ and Asif, *et al*.^34^, which illustrate that cp genomes tend to be conserved and perfectly collinear, especially in the same plant family.

### Comparative analysis of cp genomes

**Figure 3:**
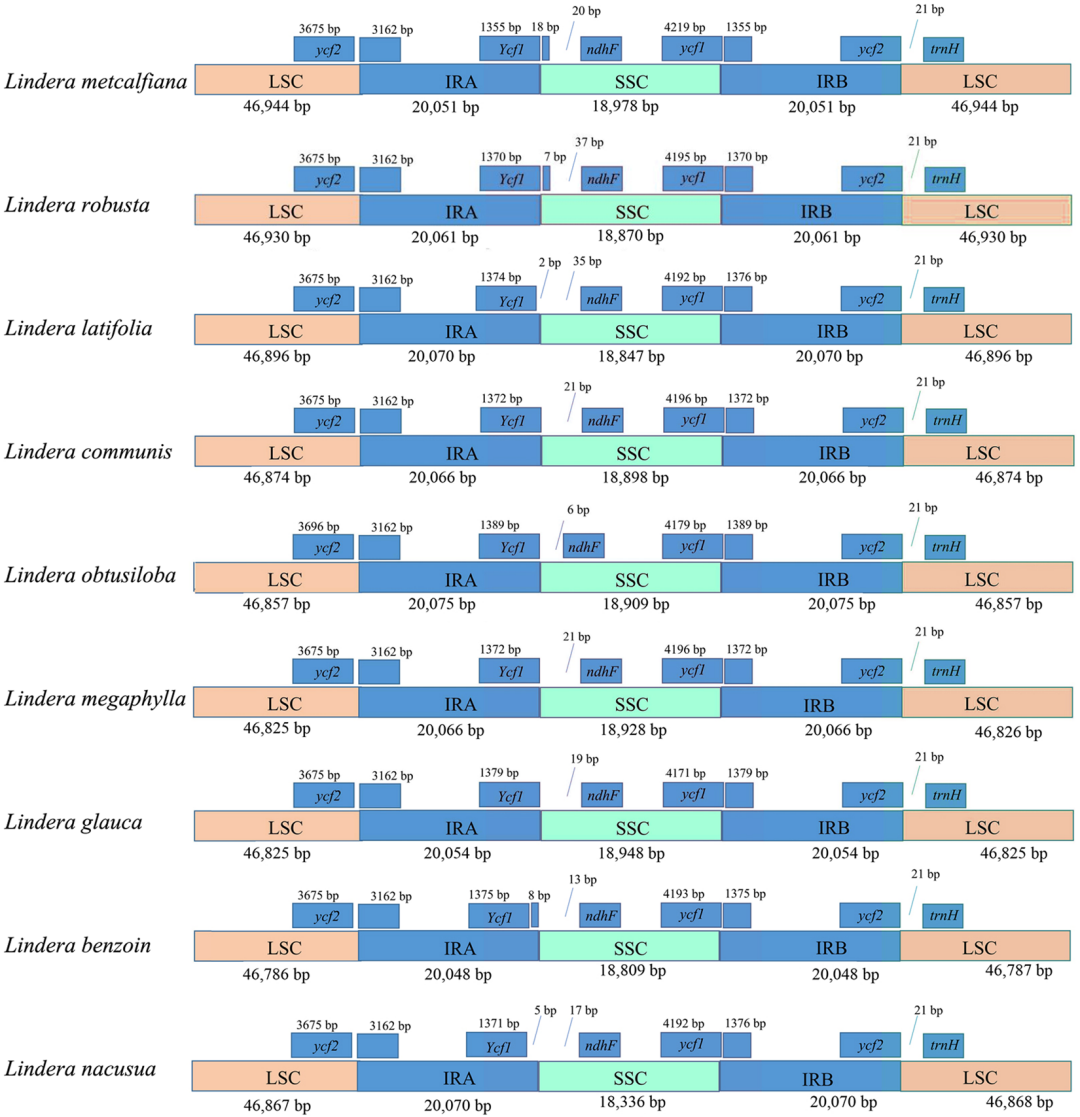
Comparison of LSC, IR, and SSC junction positions among nine *Lindera* chloroplast genomes.

Size variation in cp genomes is partly a result of contraction and expansion at the borders of the IR regions^1^. To trace the size differences among *Lindera* cp genomes, the IR-LSC and IR-SSC boundaries, with full annotations for the adjacent genes, were re-examined across nine *Lindera* cp genomes (Fig. 3). The entire *ycf1* gene crosses the SSC/IRB boundary, while another fragment of *ψycf1* is located at the IRA/SSC boundary. A *ψycf1* fragment with a length of 1,372–1,389 bp was found in the IRA region because the boundary between the SSC and IRB extended into the *ycf1* gene. In the nine *Lindera* species, the distances between *ψycf1* and *ndhF* vary from 6 (*L*. *obtusiloba*) to 37 bp (*Litsea glutinosa*) in length (Fig. 3).

### Phylogenomic analysis of sequenced Lauraceae plastomes

**Figure 4:**
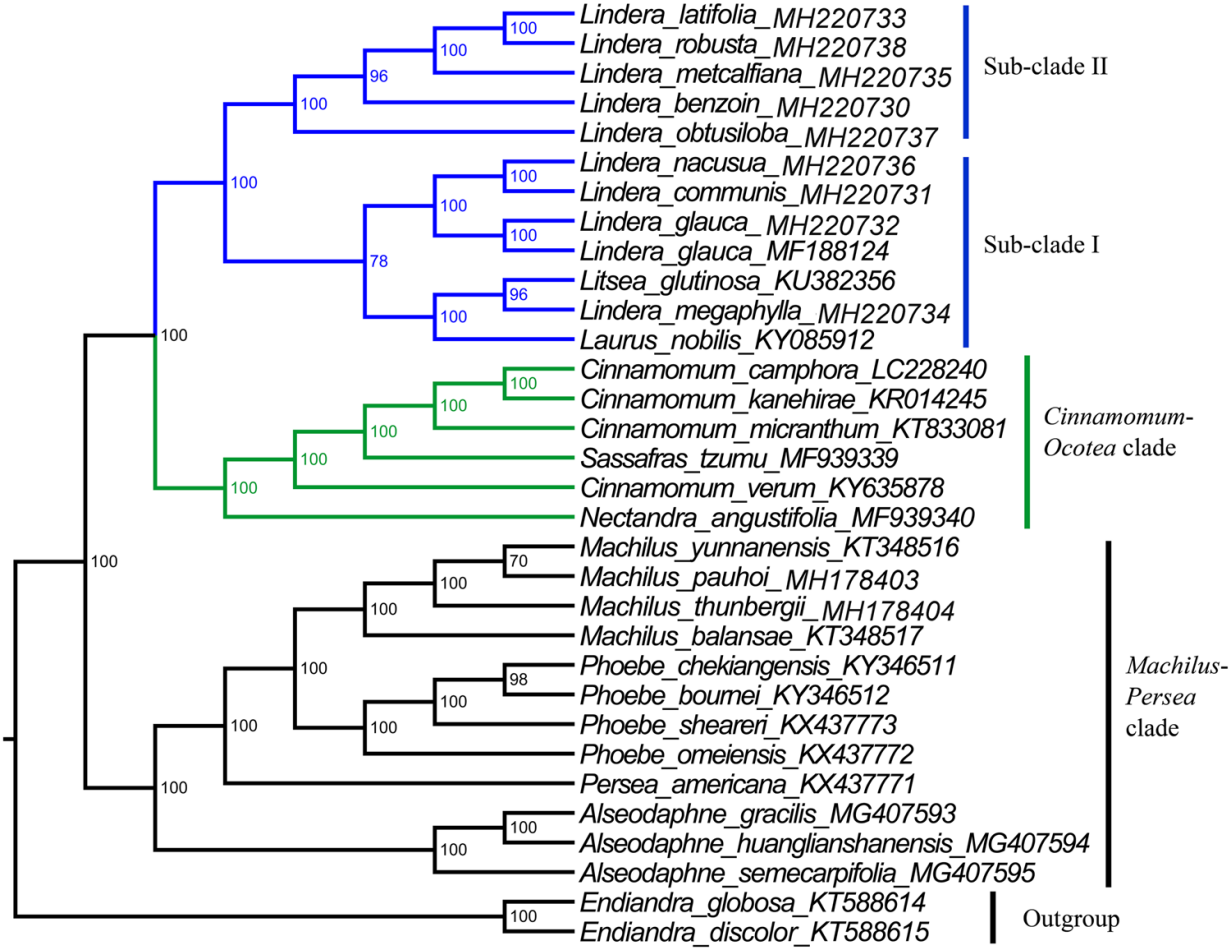
Molecular phylogenetic tree of 32 species of Lauraceae based on complete plastome sequences. Numbers at the nodes represent bootstrap percentages.

To determine the phylogenetic relationships of the nine *Lindera* species with other Lauraceae, we reconstructed a phylogenetic tree based on our nine complete cp genomes of *Lindera* and 23 fully sequenced cp genomes of related Lauraceae (Fig. 4). The tree shows that the *Lindera* species can be divided into two sub-clades. Sub-clade I (*Lindera benzoin*, *L*. *latifolia*, *L*. *metcalfiana*, *L*. *obtusiloba*, and *L*. *robusta*) is sister to sub-clade II, which contains the remaining species. Sub-clade I, however, has only 78% bootstrap support, whereas sub-clade II is 100% supported. Within sub-clade I, part I (*Lindera communis*, *L*. *glauca*, *and L*. *nacusua*) is sister to part II (*Laurus nobilis*, *Lindera megaphylla*, and *Litsea glutinosa*) (Fig. 4). These data indicate that (among the species investigated) *Laurus nobilis* and *Litsea glutinosa* are most closely related to *Lindera megaphylla*, and the *Lindera* group, which is most closely related to the *Cinnamomum*-*Ocotea* clade, comprises nine *Lindera* species, *Laurus nobilis* and *Litsea glutinosa*.

### Phylogenetic analysis of *Lindera* species

**Figure 5:**
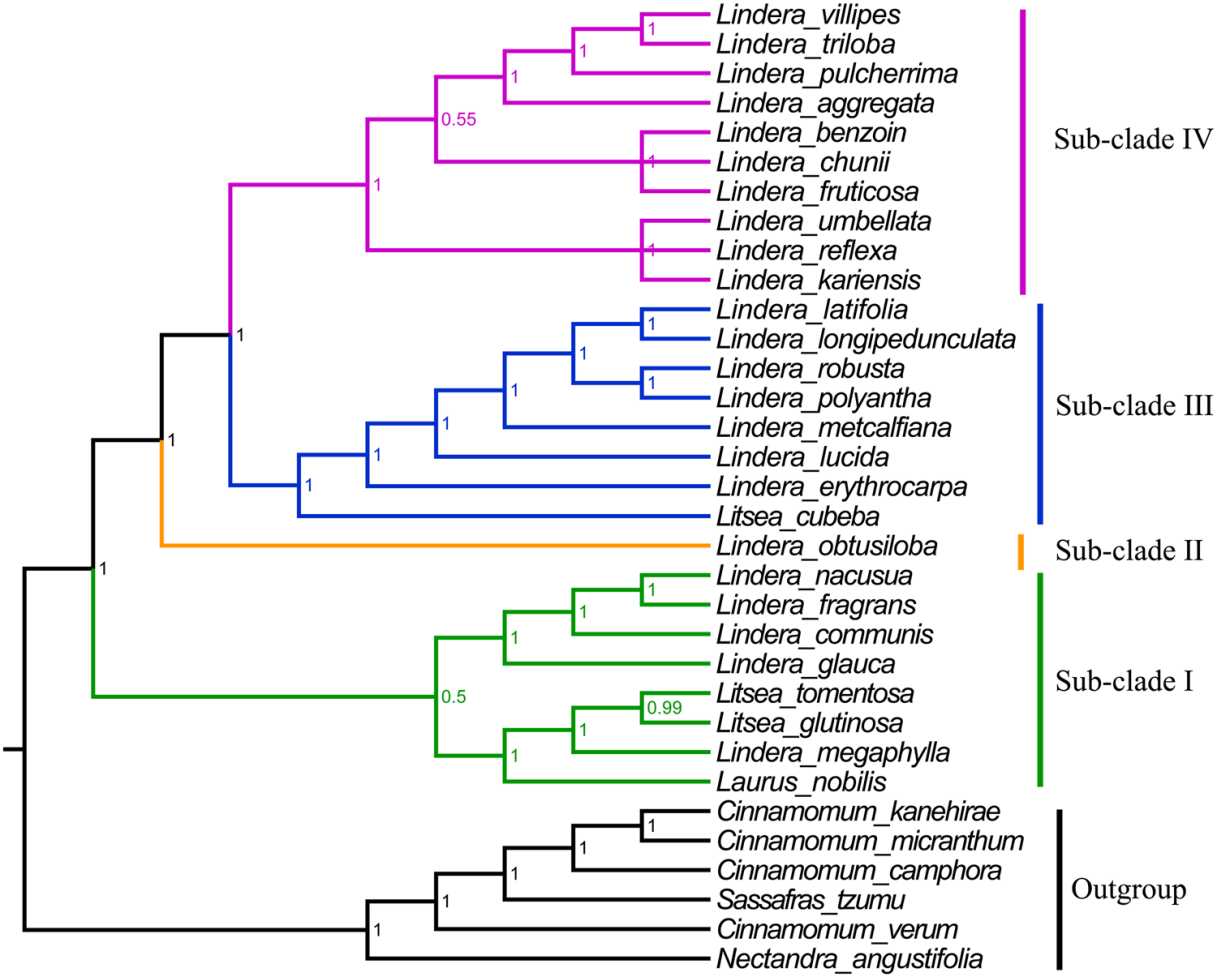
Phylogenetic relationships of 33 core Lauraceae species based on concatenated sequences of seven loci. Numbers at the nodes represent Bayesian inference posterior probabilities (BI-PP).

To better understand the phylogenetic relationships between our sequenced nine *Lindera* species and the other *Lindera* taxa with reported barcoding data, we downloaded available sequences from GenBank including *rbcL*, *matK*, *trnL*-*trnF*, *psbA*-*trnH*, *ndhF*, *ITS* and *rpb2* of all 33 core Lauraceae species (Table S1). The result of this phylogenetic analysis supports the grouping of all *Lindera* species with three *Litsea* species and *Laurus nobilis* (Fig. 5). This tree is further divided into four main sub-clades. Sub-clade I (Bayesian inference posterior probability, BI-PP = 1.00) includes *Lindera communis*, *L*. *fragrans*, *L*. *glauca*, *L*. *megaphylla*, *L*. *nacusua*, plus *Laurus nobilis*, *Lindera glutinosa*, and *Litsea tomentosa*. Sub-clade II includes only *Lindera obtusiloba*. Sub-clade III (BI-PP = 1.00) includes *Lindera erythrocarpa*, *L*. *latifolia*, *L*. *longipedunculata*, *L*. *lucida*, *L*. *metcalfiana*, *L*. *polyantha*, *L*. *robusta*, and *Litsea cubeba*. Sub-clade IV (BI-PP = 1.00) includes *Lindera aggregata*, *L*. *benzoin*, *L*. *chunii*, *L*. *fruticosa*, *L*. *kariensis*, *L*. *pulcherrima*, *L*. *reflexa*, *L*. *triloba*, *L*. *umbellata*, and *L*. *villipes*.

## Discussion

In this study, the complete cp genomes of nine *Lindera* species were sequenced using Illumina sequencing technology. These nine cp genomes possess the typical angiosperm quadripartite structure, which has a large single copy (LSC) region and a small single copy (SSC) region, separated by two short inverted repeat (IR) regions (Fig. 1). Similar to those of other sequenced Lauraceae species, the nine *Lindera* cp genomes have less length variation in the IR regions but more variation in the LSC and SSC regions. The length of the IR regions ranges from 20,048 bp in *L*. *benzoin* to 20,075 bp in *L*. *obtusiloba*. As Song, *et al*.^35^ reported, both IR regions of *Machilus balansae* and *M*. *yunnanensis* are 20,074 bp in length. The LSC region of *Lindera metcalfiana* is 93,888 bp in length, which is 315 bp larger than that of *L*. *benzoin*. The SSC region of *L*. *metcalfiana* is 18,978 bp in length, which is 642 bp larger than that of *L*. *nacusua*. A comparative analysis was conducted to explain these size differences,and the results suggest the following: the length of the entire *ycf1* gene ranges from 5,550 bp in *L*. *glauca* to 5,574 bp in *L*. *megaphylla*; the length of the truncated *ycf1* gene ranges from 1,372 bp in *L*. *communis* and *L*. *megaphylla* to 1,389 bp in *L*. *obtusiloba*; the length of the entire *ycf2* gene ranges from 6,837 bp in *L*. *metcalfiana* to 6,858 bp in *L*. *obtusiloba* (Fig. 3). It has been reported that the *ycf1* and *ycf2* genes are located in the boundaries between the IR regions and the LSC and SSC regions, and these two genes experienced incomplete duplication^35,36^. Thus, length changes in *ycf1*, *ycf2*, *ψycf1*, and *ndhF*-*ψycf1* drive the contraction and expansion of the IR regions in the cp genomes of *Lindera*.

The *ycf1* was identified as a hypervariable locus at the species level within *Lindera*, as were *ihbA*-*trnG*, *ndhA*, *ndhF*-*rpl32*, *petA*-*psbJ*, *psbK*-*psbI*, *rps16*, and *trnS*-*trnG*. At the genus level, we identified *ndhF*-*rpl32* and *ycf1* as variable regions among 29 core Lauraceae species from *Alseodaphne*, *Cinnamomum*, *Laurus*, *Lindera*, *Litsea*, *Machilus*, *Nectandra*, *Persea*, *Phoebe*, and *Sassafras*. Among these regions, *ndhF*-*rpl32*, and *ycf1* have been shown to be variable enough across seed plants for use as barcodes in plant taxonomy and phylogeny^31,35,37–40^. The four fragments *ndhF*-*rpl32*, *petA*-*psbJ*, *trnS*-*trnG*, and *ycf1*, have previously been identified as hypervariable regions in *Litsea glutinosa*, *Machilus balansae*, *M*. *yunnanensis*, *Persea americana*, *Phoebe omeiensis*, and *P*. *sheareri*^8,31,33,35^. In addition to the previous results, we found that the fragments *ihbA*-*trnG*, *ndhA*, *petA*-*psbJ*, *psbK*-*psbI*, *rps16*, and *trnS*-*trnG* seemed to be especially variable loci in *Lindera* plastomes, and they showed promising levels of variation for application in DNA barcoding or intraspecific studies.

Previously, hypervariable loci have been used as barcoding markers for taxon identification and phylogenetic analysis^3,38,41^. The chloroplast gene fragments *rbcL*, *matK*, and *psbA*-*trnH* and the nuclear internal transcribed spacer (ITS) have been reported as available markers for plant barcoding^3,29,38,41,42^. Our phylogenetic analysis using 17 complete cp genome sequences, five chloroplast regions (*rbcL*, *matK*, *trnL*-*trnF*, *psbA*-*trnH*, and *ndhF*), and two nuclear sequences (*ITS* and *rpb2*) of another 14 *Lindera* and two *Litsea* species shows that a group containing 23 *Lindera* species, three *Litsea* species, and *Laurus nobilis* was most closely related to a *Cinnamomum*-*Ocotea* clade, with strong support^11,43–45^, as in previously published phylogenetic trees. The two species *Litsea glutinosa* and *L*. *tomentosa* as well as *Laurus nobilis* are located in a sub-clade with five *Lindera* species, *Lindera communis*, *L*. *fragrans*, *L*. *glauca*, *L*. *megaphylla*, and *L*. *nacusua*, while *Litsea cubeba* was located in another sub-clade with seven *Lindera* species, *Lindera erythrocarpa*, *L*. *latifolia*, *L*. *longipedunculata*, *L*. *lucida*, *L*. *metcalfiana*, *L*. *polyantha*, *and L*. *robusta*, which is in agreement with a previous phylogenetic result by Fijridiyanto and Murakami^28^ who defined the relationships among seven *Lindera* species. And the clade containing *Laurus*, *Lindera*, and *Litsea* was the sister group of the *Cinnamomum*-*Ocotea clade* containing *Cinnamomum*, *Nectandra*, and *Sassafras* species, as found in previous studies^11,43–45^. In our study, we further determined the relationships of 16 additional *Lindera* species, *Lindera benzoin*, *L*. *chunii*, *L*. *communis*, *L*. *fragrans*, *L*. *fruticosa*, *L*. *kariensis*, *L*. *latifolia*, *L*. *longipedunculata*, *L*. *megaphylla*, *L*. *metcalfiana*, *L*. *nacusua*, *L*. *pulcherrima*, *L*. *reflexa*, *L*. *robusta*, *L*. *triloba*, and *L*. *villipes*. The results of our study are fully compatible with those of Fijridiyanto and Murakami^28^, as far as well-supported clades are concerned. A topological difference is found in the placement of *Lindera glauca*, in a clade (with *Actinodaphne* and *Neolitsea*, not examined here) that is sister to all taxa examined in both studies in Fijridiyanto and Murakami, vs. in sub-clade I here. This sub-clade, however, has practically no support.

Overall, our study reports nine chloroplast genomes of *Lindera* for the first time and compares their organizations with those of other Lauraceae species. Four divergent regions were found at the genus level, providing a valuable source of markers for future studies on species delineation and resolution of phylogenetic relationships among the Lauraceae. Our phylogenomic results also suggest that plastid phylogenomics can be regarded as a robust method for tackling difficult phylogenies and could be extended across the genera *Lindera* and *Litsea* with additional systematic sampling.

## Materials and Methods

### Plant materials

Nine *Lindera* species were used for this research. Fresh young leaves of *Lindera benzoin*, *L*. *communis*, *L*. *glauca*, *L*. *latifolia*, *L*. *megaphylla*, *L*. *metcalfiana*, *L*. *nacusua*, *L*. *obtusiloba*, and *L*. *robusta* were harvested from mature plants growing in botanical gardens (Table 3) and then immediately frozen in liquid nitrogen and stored at −80 °C. Specimens were deposited in the herbarium of the Biodiversity Research Group (BRG) of Xishuangbanna Tropical Botanical Garden, Chinese Academy of Sciences (CAS).

**Table 3:** Sampled species and their voucher specimens used in this study.

Name	Herbarium	Taxon	Voucher	Geographic origin	GenBank Accession number
*L*. *benzoin*	HITBC-BRG	*Lindera benzoin* (L.) Blume	SY34259	Beijing Botanical Garden Institute of Botany Beijing, China	MH220730
*L*. *communis*	HITBC-BRG	*Lindera communis* Hemsley	SY01432	Xishuangbanna Tropical Botanical Garden Yunnan, China	MH220731
*L*. *glauca*	HITBC-BRG	*Lindera glauca* (Siebold & Zuccarini) Blume	SY34253	Beijing Botanical Garden Institute of Botany Beijing, China	MH220732
*L. latifolia*	HITBC-BRG	*Lindera latifolia* Hook. f.	SY33219	Xishuangbanna Tropical Botanical Garden Yunnan, China	MH220733
*L*. *megaphylla*	HITBC-BRG	*Lindera megaphylla* Hemsley	SY33127	Kunming Institute of Botany Yunnan, China	MH220734
*L*. *metcalfiana*	HITBC-BRG	*Lindera metcalfiana* var. dictyophylla (C. K. Allen) H. P. Tsui	SY34595	Xishuangbanna Tropical Botanical Garden Yunnan, China	MH220735
*L*. *nacusua*	HITBC-BRG	*Lindera nacusua* (D. Don) Merr.	SY34537	South China Botanical Garden Guangzhou, China	MH220736
*L*. *obtusiloba*	HITBC-BRG	*Lindera obtusiloba* Blume	SY34165	Beijing Botanical Garden Institute of Botany Beijing, China	MH220737
*L*. *robusta*	HITBC-BRG	*Lindera robusta* (C. K. Allen) H. P. Tsui	SY34225	South China Botanical Garden Guangzhou, China	MH220738

### DNA preparation and chloroplast sequencing

The cetyltrimethylammonium bromide (CTAB) method was used to extract total genomic DNA. Next-generation sequencing was performed according to Yang, *et al*.^46^, and nine universal primer pairs from their study were also taken to perform long-range PCR. Then, the PCR products were purified and combined. Following the manufacturer’s instructions (Illumina Nextera XT library), the mixture was fragmented and used to construct 500-bp short-insert libraries. All nine complete *Lindera* cp genomes were sequenced using a Genome Analyzer (Illumina HiSeq2000) at the Germplasm Bank of Wild Species, Kunming Institute of Botany, CAS.

### Cp genome assembly and annotation

All nine *Lindera* raw sequencing reads were filtered using the NGS QC Tool Kit to obtain high-quality short reads. Then, the raw reads were trimmed according to their quality, removing bases from the 5′ and 3′ ends until no base with Q < 20 was found. After that, the chloroplast genome was de novo assembled using the trial version of CLC v.8 (http://www.qiagenbioinformatics.com). The contigs were aligned using the publicly available cp genome of *Litsea glutinosa*^8^ in Geneious 4.8 (http://www.geneious.com/) as a reference. Dual Organellar GenoMe Annotator (DOGMA, http://dogma.ccbb.utexas.edu/) software was used to annotate the cp genomes and identify genes encoding proteins^47^, transfer RNAs (tRNAs), and ribosomal RNAs (rRNAs). The OrganellarGenomeDRAW tool (OGDRAW, http://ogdraw.mpimp-golm.mpg.de/) was used to draw the genome maps of *Lindera benzoin*, *L*. *communis*, *L*. *glauca*, *L*. *latifolia*, *L*. *megaphylla*, *L*. *metcalfiana*, *L*. *nacusua*, *L*. *obtusiloba*, and *L*. *robusta*.

### Sliding window analysis to identify hypervariable regions

MAFFT (http://mafft.cbrc.jp/alignment/server) was used to align the nine cp genomes with one another. Afterward, we manually adjusted these sequences using BioEdit software (http://www.mbio.ncsu.edu/bioedit/bioedit.html). DnaSP version 5.0 with a sliding window analysis was used to calculate the nucleotide variability values (π) within the chloroplast genomes. The window length was set to 600 bp and the step size to 200 bp. Then, the R program was used to plot values. In addition, we used this method to analyse the hypervariable regions among the nine *Lindera* cp genomes and those of *Alseodaphne gracilis* (GenBank accession number MG407593)^48^, *A*. *huanglianshanensis* (GenBank accession number MG407594)^48^, *A*. *semecarpifolia* (GenBank accession number MG407595)^48^, *Cinnamomum camphora* (GenBank accession number LC228240)^49^, *C*. *kanehirae* (GenBank accession number KR014245)^50^, *C*. *micranthum* (GenBank accession number KR014245)^50^, *C*. *verum* (GenBank accession number KY635878)^9^, *Laurus nobilis* (GenBank accession number KY085912)^13^, *Lindera glauca* (GenBank accession number MF188124), *Litsea glutinosa* (GenBank accession number KU382356)^8^, *Machilus balansae* (GenBank accession number KT348517)^35^, *M*. *pauhoi* (GenBank accession number MH178403), *M*. *thunbergii* (GenBank accession number MH178404), *M*. *yunnanensis* (GenBank accession number KT348516)^35^, *Nectandra angustifolia* (GenBank accession number MF939340)^13^, *Persea americana* (GenBank accession number KX437771)^31^, *P*. *chekiangensis* (GenBank accession number KY346511), *Phoebe omeiensis* (GenBank accession number KX437772)^31,32^, *P*. *sheareri* (GenBank accession number KX437773)^31,32^, *P*. *zhennan* (GenBank accession number KY346512), and *Sassafras tzumu* (GenBank accession number MF939339)^13^.

### Phylogenetic analyses

The plastome sequences of *Alseodaphne gracilis*, *A*. *huanglianshanensis*, *A*. *semecarpifolia*, *Cinnamomum camphora*, *C*. *kanehirae*, *C*. *micranthum*, *C*. *verum*, *Endiandra discolor* (GenBank accession number KT588615)^51^, *E*. *globosa* (GenBank accession number KT588614)^51^, *Laurus nobilis*, *Lindera glauca*, *Litsea glutinosa*, *Machilus balansae*, *M*. *pauhoi*, *M*. *thunbergii*, *M*. *yunnanensis*, *Nectandra angustifolia*, *Persea americana*, *Phoebe chekiangensis*, *P*. *omeiensis*, *P*. *sheareri*, *P*. *zhennan*, and *Sassafras tzumu* were downloaded from the NCBI GenBank. Thereafter, we used MAFFT to align these 23 sequences and our nine *Lindera* sequences and manually edited where necessary with BioEdit software. After these steps, the jModelTest 2.0 program was used to calculate nucleotide substitution, and the results indicated that the optimal model was “GTR + G”^52^. The “GTR + G” model was used for all maximum likelihood (ML) analyses implemented in RAxML version 8.0.20, as suggested in the manual^53^. Nonparametric bootstrapping was performed with the “fast bootstrap” algorithm of RAxML and 1000 replicates. The cp genomes of *Endiandra discolor* and *E*. *globosa* were used as the outgroup.

We assembled sequence alignments for 33 taxa of Lauraceae, 17 (*Cinnamomum camphora*, *C*. *kanehirae*, *C*. *micranthum*, *C*. *verum*, *Laurus nobilis*, *Lindera benzoin*, *L*. *communis*, *L*. *glauca*, *L*. *latifolia*, *L*. *megaphylla*, *L*. *metcalfiana*, *L*. *nacusua*, *L*. *obtusiloba*, *L*. *robusta*, *Litsea glutinosa*, *Nectandra angustifolia*, and *Sassafras tzumu*) with complete cp genome sequences, plus ITS and *rpb2* sequences, and 14 *Lindera* species (*Lindera aggregata*, *L*. *chunii*, *L*. *erythrocarpa*, *L*. *fragrans*, *L*. *fruticosa*, *L*. *kariensis*, *L*. *longipedunculata*, *L*. *lucida*, *L*. *polyantha*, *L*. *pulcherrima*, *L*. *reflexa*, *L*. *triloba*, *L*. *umbellata*, and *L*. *villipes*), and two *Litsea* species (*Litsea cubeba* and *L*. *tomentosa*) with the DNA sequences of *rbcL*, *matK*, *trnL*-*trnF*, *psbA*-*trnH*, *ndhF*, ITS^54^ and *rpb2* from GenBank (Table S1). MAFFT software was used to analyse these sequences, and incongruous sequences of the same species were removed. The seven-sequence matrix was then manually adjusted, and Sequencher 4.10 (http://www.genecodes.com) was used to merge identical sequences. Then, a joint matrix was constructed using Sequence Matrix v.1.7.8, and jModelTest 2.0 was used to calculate the nucleotide substitution. The optimal model was chosen (“GTR + I + G”) (freqA = 0.3003, freqC = 0.1988, freqG = 0.1919, freqT = 0.3090, R(a) [AC] = 0.8998, R(b) [AG] = 2.0890, R(c) [AT] = 0.2648, R(d) [CG] = 0.4178, R(e) [CT] = 1.9183, R(f) [GT] = 1.0000, p-inv = 0.7020, gamma shape = 0.3000) to construct the phylogenetic tree^52^. Phylogenetic relationships were reconstructed using Bayesian inference (BI) and ML methods in MrBayes version 3.1.2^55^.

### Data archiving statement

The complete cp genome sequence data of the nine *Lindera* taxa have been submitted to the GenBank of NCBI. The GenBank accession numbers are MH220730 (*Lindera benzoin*), MH220731 (*L*. *communis*), MH220732 (*L*. *glauca*), MH220733 (*L*. *latifolia*), MH220734 (*L*. *megaphylla*), MH220735 (*L*. *metcalfiana*), MH220736 (*L*. *nacusua*), MH220737 (*L*. *obtusiloba*), and MH220738 (*L*. *robusta*).

## Electronic supplementary material

Supplementary information

Dataset 1
