# Supplementary material for: Comparative chloroplast genomics and phylogenetics of nine Lindera species (Lauraceae)
Source: Sci Rep. 2018 Jun 11;8:8844. doi: 10.1038/s41598-018-27090-0 (PMC5995902; doi:10.1038/s41598-018-27090-0)
Supplement: Supplementary file 1 — Supplementary information [file 41598_2018_27090_MOESM1_ESM.pdf]

Supplementary information

Comparative chloroplast genomics and phylogenetics of nine *Lindera* species (Lauraceae)

Mei-Li Zhao[2,3] zhaomeili@xtbg.ac.cn

Yu Song[1,4,*] songyu@xtbg.ac.cn

Jun Ni[2] nijun@ipp.ac.cn

Xin Yao[1] yaoxin@xtbg.org.cn

Yun-Hong Tan[1,4] tyh@ xtbg.org.cn

Zeng-Fu Xu[2,*] zfxu@xtbg.ac.cn

[1] Center for Integrative Conservation, Xishuangbanna Tropical Botanical Garden, Chinese Academy of Sciences, Menglun, Mengla, Yunnan 666303, China

[2] Key Laboratory of Tropical Plant Resources and Sustainable Use, Xishuangbanna Tropical Botanical Garden, Chinese Academy of Sciences, Menglun, Mengla, Yunnan 666303, China

[3] College of Life Sciences, University of Chinese Academy of Sciences, Beijing 100049, China

[4] Southeast Asia Biodiversity Research Institute, Chinese Academy of Sciences, Yezin, Nay Pyi Taw, Myanmar

* Corresponding authors (Email: songyu@xtbg.ac.cn and zfxu@xtbg.ac.cn)

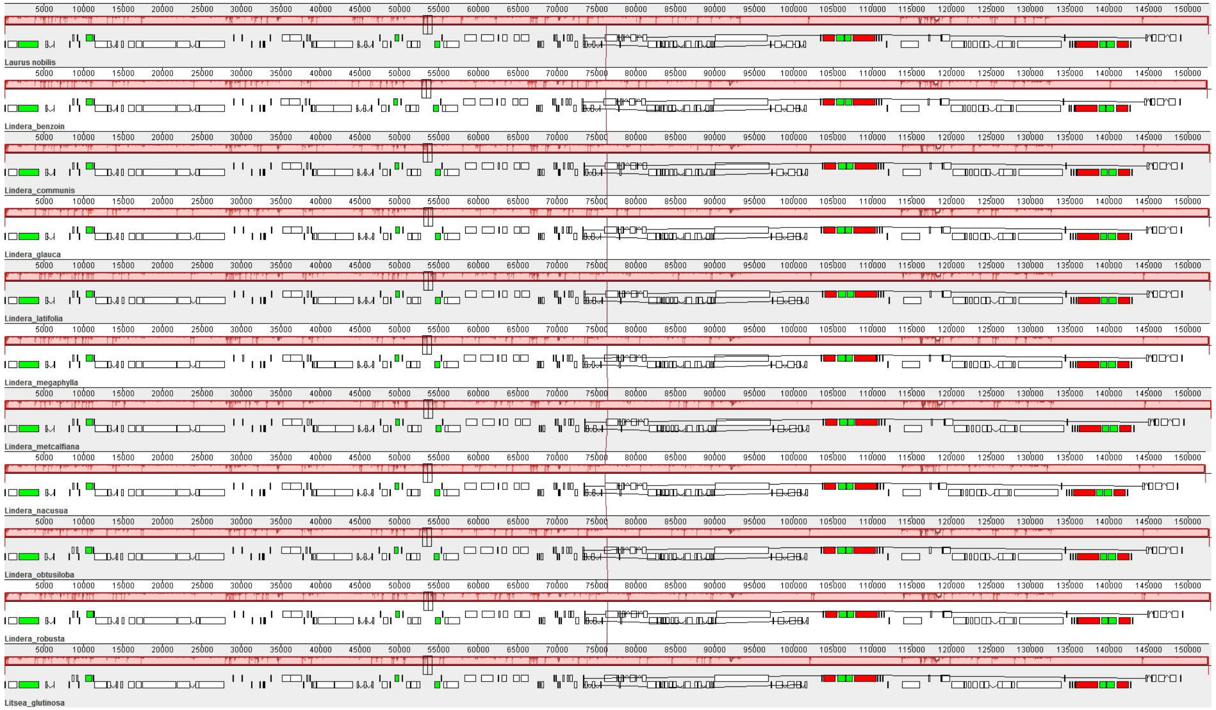

Figure S1 Synteny and rearrangements detected in *Lindera*, *Laurus nobills* and *Litsea glutinosa* chloroplast genome sequences using the Mauve multiple-genome alignment program. Representative results from the analysis of eleven species are shown. Coloured bars indicate syntenic blocks, and connecting lines indicate correspondences between blocks.

Table S1 GenBank accession numbers for DNA sequences of *rbcL*, *matK*, *trnL-trnF*, *psbA-trnH*, *ndhF*, *ITS* and *rpb2* from 33 core Lauraceae species.
